# A Hypothetical Model of Crossing *Bombyx mori* Nucleopolyhedrovirus through Its Host Midgut Physical Barrier

**DOI:** 10.1371/journal.pone.0115032

**Published:** 2014-12-12

**Authors:** Yang Cheng, Xue-Yang Wang, Hao Hu, Nabil Killiny, Jia-Ping Xu

**Affiliations:** 1 School of Life Sciences, Anhui Agricultural University, Hefei, People's Republic of China; 2 Department of Entomology and Nematology, Citrus Research and Education Center, IFAS, University of Florida, Lake Alfred, Florida, United States of America; Wuhan Bioengineering Institute, China

## Abstract

*Bombyx mori* nucleopolyhedrovirus (BmNPV) is a primary pathogen of silkworm (*B. mori*) that causes severe economic losses each year. However, the molecular mechanisms of silkworm-BmNPV interactions, especially the silkworm proteins that can interact with the virus, are still largely unknown. In this study, the total and membrane proteins of silkworm midguts were displayed using one- and two-dimensional electrophoresis. A virus overlay assay was used to detect *B. mori* proteins that specifically bind to BmNPV particles. Twelve proteins were located and identified using mass spectrometry, and the different expression of the corresponding genes in BmNPV susceptible and resistant silkworm strains also indicated their involvement in BmNPV infection. The 12 proteins are grouped based on their potential roles in viral infection, for example, endocytosis, intracellular transportation, and host responses. Based on these results, we hypothesize the following: I) vacuolar ATP synthase catalytic subunit A and subunit B may be implicated in the process of the membrane fusion of virus and the release of the nucleocapsid into cytoplasm; II) actin, enolase and phosphoglycerate kinase are cytoskeleton associated proteins and may play an important role in BmNPV intracellular transportation; III) mitochondrial prohibitin complex protein 2, ganglioside-induced differentiation-associated protein, calreticulin, regucalcin-like isoform X1 and 60 kDa heat shock protein are involved in cell apoptosis regulation during BmNPV infection in larvae midguts; IV) ribosomal P0 may be associated with BmNPV infection by regulating gene expression of BmNPV; V) arginine kinase has a role in the antiviral activities against BmNPV. Our work should prove informative by providing multiple protein targets and a novel direction to investigate the molecular mechanisms of the interactions between silkworms and BmNPV.

## Introduction

The silkworm, *Bombyx mori* L. (Lepidoptera: Bombycidae), is an economically important insect for production of silk and recombinant proteins, and also a good model of the Lepidoptera [1]. *Bombyx mori* nucleopolyhedrovirus (BmNPV) is a primary pathogen of the domestic silkworm, and always causes severe economic loss [2].

In the NPV replication cycle, there are two different virion phenotypes, which are the occlusion-derived virus (ODV) and the budded virus (BV) [3]. BVs infect a broad range of cell types and transmit virus among insect tissues within an infected larva, whereas ODVs are contained in polyhedrons and form occlusion bodies (OBs) which infect only columnar epithelial cells of the insect midguts and are required for the oral transmission of virus between insect hosts [4], [5].

At present, the molecular interaction mechanisms between BmNPV and *B. mori* remain unclear. Studies on the functions of anti-viral proteins isolated from *B. mori* were reported frequently in the last ten years. It has been reported that *B. mori* serine protease-2, lipase-1 and alkaline trypsin protein purified from the digestive juice of *B. mori* larvae showed strong antiviral activity to BmNPV in vitro [6]–[8]. Using the fluorescent differential display (FDD) technique, Bms3a was found related to BmNPV resistance in silkworm [9]. But the roles of these proteins in the process of BmNPV infection or *B. mori* anti-infection are not stated, meanwhile, studies on the interactions between BmNPV and *B. mori* at the system-wide level of larva using the methodology of far-western blot and mass spectrometry have not been reported yet. Far-western blot, also known as virus overlay assay, has been used successfully to detect proteins that are potential virus receptors in the body of insect vectors [10]. Initially, several proteins within *Myzuspersicae* were found to bind to potato leaf roll virus (PLRV) in vitro [11]. Since then, many virus-binding proteins were determined in host insects. Kikkert *et al*. found a 94-kDa thrips protein that exhibited specific binding to tomato spotted wilt virus (TSWV) particles in *Frankliniella occidentalis* and *Thrips tabaci* using virus overlay assays [12]. Bandla *et al* also found out that a 50-kDa protein in larval midguts of *F. occidentalis* exhibited interaction with TSWV using similar method almost at the same time [13]. But the virus-binding proteins were not identified exactly. The development of mass spectrometry in recent years makes the identification of proteins feasible. For example, 5 proteins of *Laodelphax striatellus* exhibited interactions with Rice stripe virus (RSV) using virus overlay assays and they were identified using Nano LC-ESI-CID-MS/MS analysis [10].

In our study, virus overlay assays were performed in the screening for BmNPV binding proteins from larval midguts of *B. mori*. The results showed that twelve proteins of *B. mori* bound specifically to purified BmNPV particles *in vitro*, and these potential proteins were identified using MALDI-TOF/TOF MS analysis. The potential functions of these binding proteins were further investigated and speculated reasonably.

## Results

### Interaction between BmNPV particles and silkworm midgut proteins

**Figure 1:**
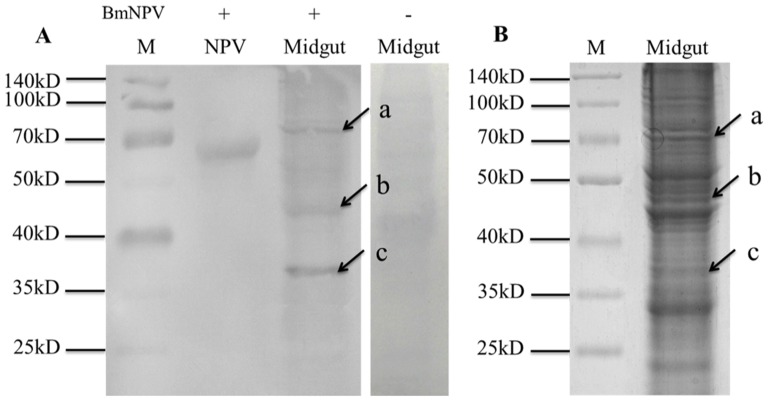
Virus binding experiment on total proteins of *B. mori* midgut resolved by SDS-PAGE. A) Virus overlay binding experiment. B) Separation of *B. mori* midgut total proteins by SDS-PAGE. M indicated the standard prestained protein molecular weight marker (Thermo Scientific), NPV was set as positive control, Midgut referred to *B. mori* midgut total proteins separated by SDS-PAGE, and the right lane in A) was the negative control that overlaid in binding buffer without BmNPV particles before incubated with monoclonal antibodies against baculovirus gp64. The plus and minus signs on the top meant membranes incubated with or without BmNPV particles. Arrows named a, b, c in A) and B) referred to the detected bands on PVDF membrane and the corresponding proteins in gel.

**Figure 2:**
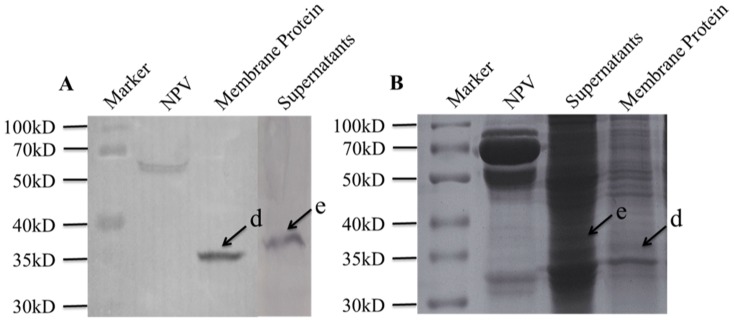
Virus binding experiment on lipid-associated and hydrophilic proteins of *B. mori* midgut resolved by SDS-PAGE. A) Virus overlay binding experiment. B) Separation of *B. mori* midgut lipid-associated and hydrophilic proteins by SDS-PAGE. M indicated the standard prestained protein molecular weight marker, NPV was set as positive control, Supernatants meant hydrophilic proteins. Arrows named d, e in A) and B) referred to the detected bands on PVDF membrane and the corresponding proteins in gel.

Virus overlay assays were applied to ascertain whether specific binding of BmNPV particles to *B. mori* midgut total proteins or lipid-associated midgut membrane proteins would occur. Total proteins were characterized by 12% SDS-PAGE followed by far-Western blot experiment, and three clear bands indicated by arrows were detected (Fig. 1A). In order to determine the three protein bands, a parallel SDS-PAGE assay was performed (Fig. 1B), and three protein bands named a, b and c, respectively, were cut out for MALDI-TOF/TOF MS analysis. Similar experiments were performed to both lipid-associated membrane proteins and hydrophilic proteins, and the results were shown in Fig. 2A and 2B. Two protein bands (a lipid-associated one and a hydrophilic one) named d and e, respectively, were cut out for further analysis.

**Figure 3:**
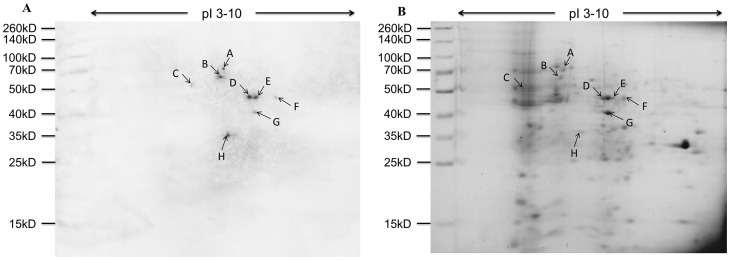
Virus binding experiment on total proteins of *B. mori* midgut resolved by 2-DE. A) Virus overlay binding experiment. B) Separation of *B. mori* midgut total proteins by 2-DE. Molecular mass was indicated on the left and isoelectric point (PI) range on the top. Arrows named A to H in A) and B) referred to the detected spots on PVDF membrane and the corresponding proteins in gel, respectively.

Total proteins were also characterized by 2-DE followed by far-Western blot experiment, and eight spots were visualized clearly and indicated by arrows named A to H respectively (Fig. 3A). The eight corresponding spots in gel were also indicated by arrows in Fig. 3B and cut out for MALDI-TOF/TOF MS analysis.

### Identification of the BmNPV binding proteins

Comparisons between the stained electrophoretic profiles of *B. mori* proteins in the gel and results of far-Western blot experiments on the membrane allowed the unambiguous selection of protein bands or spots from the gels (indicated by arrows in Fig. 1B, 2B and 3B) for MALDI-TOF/TOF MS analysis. Identified proteins were listed in Table 1 and Table 2. The listed proteins in Table 1 were separated by SDS-PAGE, including vacuolar ATP synthase catalytic subunit A (ATP-A), actin, ribosomal P0 protein (RP0), mitochondrial prohibitin complex protein 2 (PHB2) and ganglioside-induced differentiation associated protein (GDAP). In Table 2, eight different proteins were separated by 2-DE, which included vacuolar ATP synthase catalytic subunit A (ATP-A),60 kDa heat shock protein (HSP), vacuolar ATP synthase subunit B (ATP-B), calreticulin (CRT), enolase (En), phosphoglycerate kinase (PGK), arginine kinase (AK), regucalcin-like isoform X1 (RCX1). The relative molecular weights (MW) calculated in the NCBI database for these twelve proteins were in good agreement with the experimentally observed values.

**Table 1:** Identification of *B. mori* proteins separated by SDS-PAGE.

Bands	Identified proteins	Score	Coverage	NCBI code	Mw (kDa)
a	Vacuolar ATP synthase catalytic subunit A, *Bombyx mori* (ATP-A)	1715	43%	gi|148298878	68.558
b	Actin, *Anopheles gambiae*	952	39%	gi|158293921	42.703
c	Ribosomal P0 protein, *Bombyx mori* (RP0)	222	18%	gi|37359627	34.197
d	Mitochondrial prohibitin complex protein 2, *Bombyx mori* (PHB2)	66	8%	gi|114051710	33.212
e	Ganglioside-induced differentiation-associated-protein, *Bombyx mori* (GDAP)	125	13%	gi|315633209	38.479

**Table 2:** Identification of *B. mori* proteins separated by 2-DE.

Spots	Identified proteins	Score	Coverage	NCBI code	Mw (kDa)
A	Vacuolar ATP synthase catalytic subunit A, *Bombyx mori* (ATP-A)	221	8%	gi|148298878	68.558
B	60 kDa heat shock protein, *Bombyx mori* (HSP60)	102	6%	gi|512896628	61.193
C	Vacuolar ATP synthase subunit B, *Bombyx mori* (ATP-B)	128	7%	gi|148298717	54.667
D	Calreticulin, *Bombyx mori* (CRT)	66	4%	gi|28804517	46.082
E	Enolase, *Bombyx mori* (En)	717	24%	gi|148298800	47.164
F	Phosphoglycerate kinase, *Bombyx mori* (PGK)	137	11%	gi|512916352	44.518
G	Arginine kinase, *Bombyx mori* (AK)	606	30%	gi|112983926	40.308
H	Regucalcin-like isoform X1, *Bombyx mori* (RCX1)	206	18%	gi|512924941	35.658

The bands and spots numbers corresponded to the numbers given in Fig. 1, Fig. 2 and Fig. 3. Protein bands and spots were subjected to in-gel trypsin digestion. Protein fragments were then analyzed by MALDI-TOF/TOF MS analysis on ABI 4800 Plus MALDI TOF/TOF Analyzer. The peptide sequences obtained from MALDI-TOF/TOF MS were searched against the protein sequences from NCBInrmetazoa using the Mascot algorithm (http://www.matrixscience.com). Protein identification was accepted when the matching scores were significant at P<0.05, as based on the Mowse score (Matrix Science, London, UK).

### Expression analysis of virus-binding proteins

In order to study the roles of these virus-binding proteins in the infection process by BmNPV, the relative expression levels of the corresponding genes in larval midgut of susceptible *B. mori* strain P50 with or without virus challenge were examined by real-time qPCR. The resistant strain A35 was also included for comparison. The primer sequences were listed in Table 3.

**Table 3:** Primers for real-time PCR.

Target gene	GenBank accession number	Primer sequence (5'→3')	Length of product
*ATP-A*	NM_001098359	Forward: AGTTCAAAATGGCGAGCAAAG	96
		Reverse: CGACGGGTCCAGATACGG	
*ATP-B*	NM_001098358	Forward: GCCGTGGTAGGTGAGGAGG	145
		Reverse: TGGGGAAGATACGCAGCAAC	
*PHB2*	NM_001046861	Forward: TGAAAGGGCAAAGCAAGAGC	175
		Reverse: GAAGACACGGTTCTGAGATTGAG	
*GDAP*	NM_001199937	Forward: CCTGTCGGTAATTGGTTATGCTG	231
		Reverse: AACTGGAGGTGGAGCCGTA	
*HSP60*	XM_004923900	Forward: TGGCTATTGCTACTGGTGGAG	147
		Reverse: ATTTCTTACCCTTGCCCTTCA	
*Crt*	NM_001043610	Forward: ATTTGTGGCAAGTCAAGTCCG	221
		Reverse: CGCTGCGTCTCCAGTCTCA	
*En*	NM_001098361	Forward: TCGCACCAAACATACAAAACAAC	116
		Reverse: GAACTCAGAGGCGGCTACATC	
*PGK*	XM_004928563	Forward: CTGCTGGCGTGTTTGAGTTTG	156
		Reverse: GCGAGACCTTGTCCTCTGTTC	
*AK*	NM_001043937	Forward: GTGGACACGCTCGGCAAC	221
		Reverse: TGCTGCTGGGTCTCCTTCG	
*RCX1*	XM_004930659	Forward: ATTTCGTGGTGGGTCTGGA	122
		Reverse: GTTGAATCTATTGTTGGGGTTGTC	
*Actin*	NM_001126253	Forward: TACGAAGGTTACGCTCTGCCC	138
		Reverse: GTCACGAACGATTTCCCTCTCA	
*RP0*	NM_001043658	Forward: GTGGCTCCAGTATCGTGCTC	131
		Reverse: GGTGAACACGAAGCCAACG	
*RPS3*	NM_001043788	Forward: CGATTCAACATTCCAGAGCA	142
		Reverse: GAACACCATAGCAAGCACGAC	

**Figure 4:**
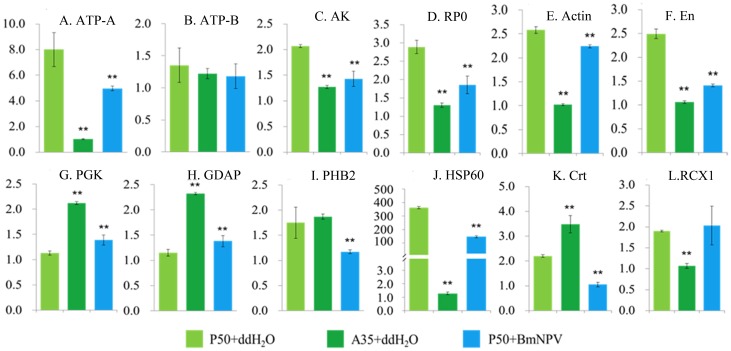
Real-time PCR analysis of expression profiles of BmNPV binding proteins in *B. mori* midguts. Columns with different colors indicated the experimental treats to the larvae. A to L refer to relative expression level of ATP-A, ATP-B, AK, RP0, Actin, En, PGK, GDAP, PHB2, HSP60, Crt, and RCX1 respectively. Data were normalized using*Bmrps3*and represented as means±standard errors of the means from three independent experiments. ** Indicates statistical significance<0.01(ANOVA and LSD aposteriori test).

Real-time qPCR analysis was performed between BmNPV infected P50 larvae and the control group treated with ddH_2_O. Fifth instar molt larvae of P50 were starved overnight and fed with 500 OB of BmNPV T3 strain or ddH_2_O per larva orally. The relative expression levels of *PGK* (Fig. 4G) and *GDAP* (Fig. 4H) in P50 midguts were up-regulated significantly (*P*<0.01) at 48hpi, while the levels of eight genes, *ATP-A* (Fig. 4A), *AK* (Fig. 4C), *RP0* (Fig. 4D), *actin* (Fig. 4E), *En* (Fig. 4F), *PHB2* (Fig. 4I), *HSP* (Fig. 4J), *Crt* (Fig. 4K), respectively, were down-regulated significantly (*P*<0.01) at 48hpi. The relative expression levels of *ATP-B* (Fig. 4B) and *RCX1* (Fig. 4L) were not significantly different between the infected and the control.

The relative expression levels of most examined genes, except *ATP-B* (Fig. 4B) and *PHB2* (Fig. 4I), were significantly different between P50 and A35 treated with ddH_2_O. The relative expression levels of seven examined genes in P50, *ATP-A* (Fig. 4A), *AK* (Fig. 4C), *RP0* (Fig. 4D), *Actin* (Fig. 4E), *En* (Fig. 4F), *HSP* (Fig. 4J), *RCX1* (Fig. 4L) respectively, were significantly higher than those in A35 (*P*<0.01), while the levels of three examined genes in P50, *PGK* (Fig. 4G), *GDAP* (F. 4H), *Crt* (Fig. 4K), respectively, were significantly lower than those in A35 (*P*<0.01).

## Discussions

The molecular mechanisms of the interaction between *B. mori* and BmNPV are still unclear at present, illuminating the mechanisms is of significant importance not only in biological research but also in profit increase of sericulture. To this end, we designed an experimental program to study the *B. mori* proteins involved in the infection process by BmNPV.

It is well known that baculovirus budded viruses (BVs) enter host cell via clathrin-mediated endocytosis [14]. Once inside the endosome, the virus encoded gp64 protein can be enabled by the acidic environment to promote membrane fusion of the virusand endosome to release the nucleocapsid into cytoplasm, thus it is necessary forviral infection to occur [15]–[17]. Vacuolar ATP synthase (or V-ATPase), originally identified in intracellular compartments, such as endosomes, lysosomesand thecentral vacuoles of fungi and plants (hence the name), is the most important transport protein for pH regulation of the intracellular compartments [17]–[19]. Based on previous research, V-ATPase facilitated the infection of baculovirus by acidifying the endosomes [15], which indicates that BmNPV-susceptible strain may have a higher V-ATPase expression level. In our study, the relative expression level of V-ATPase subunit A in P50 was about 8 fold of that in A35 (Fig. 4A), and the down-regulation of V-ATPase subunit A at 48 hpi might be due to the activation of the host immune system to suppress the infection. The relative expression level of V-ATPase subunit B was not significantly different in the three groups (Fig. 4B), indicating that subunit A was more important in BmNPV infection.

Cytoskeleton is important for maintenance of cell shape, cell motility and intracellular transport [21], and it is generally thought that viruses need cytoskeleton during infection [20]. Here the interaction of several cytoskeleton-associated proteins with BmNPV was determined, and they were actin, enolase (En) and phosphoglycerate kinase (PGK). It is known that actin is the major component of microfilaments [22], and En and PGK are enzymes involved in glycolysis initially [23], [24]. Recent studies demonstrated that these two enzymes had their roles in diseases and immune responses [25], [26]. Boone et al. reported that PGK could bind to actin and plasminogen [27]. Plasminogen is the zymogen of plasmin, a key component of the fibrinolytic system, in which plasminogen plays two main functions of blood clot dissolution and extracellular matrix disintegration [28]. En has been found on the tegument surface of *Schistosomabovis*, where it acts as a plasminogen receptor to avoid blood clot formation and facilitates the infection of the host [28], [29]. In our study, a significant lower relative expression level of En in A35 (Fig. 4F) indicates its role in virus infection.

In lieu of an adaptive immune system, apoptosis plays a central role in regulating cellular or environmental stimuli in Lepidopteran insect cells during virus infection [30], where larvae resist baculovirus infection by selective apoptosis of the infected cells from midguts epithelium and by sloughing off the infected cells [31]. Using virus overlay assay, five proteins including mitochondrial prohibitin complex protein 2 (PHB2), ganglioside induced differentiation associated protein (GDAP), Calreticulin (CRT), Regucalcin-like isoform X1 (RCX1) and 60 kDa heat shock protein (HSP60) of *B. mori* were identified interacting with BmNPV in our study. Previous researches have revealed their functions in apoptosis. It was reported that PHB2 inhibited apoptosis and regulated the mitochondrial morphology by interacting with HAX-1 [32], [33]; CRT could be induced to form a complex with gC1qR and prevented apoptosis [34]; overexpression of regucalcin had a suppressive effect on cell death and apoptosis induced by various factors in cloned normal rat kidney proximal tubular epithelial NRK52E cells [35]. Based on their role of apoptosis suppression, hosts needed to decrease the expression of these proteins to promote apoptosis and prevent virus infection when exposed to virus, and this explained the notable down-regulation of PHB2 and CRT at 48 hpi (Fig. 4I and K). Gao et al. reported that higher expression level of BmGDAP in *B. mori* midguts during BmCPV infection could activate the apoptosis and death programs of infected cells by accelerating the mitochondrial division [36]. We determined the binding of BmGDAP and BmNPV, and found its similar expression level in *B. mori* midguts during BmNPV infection (Fig. 4H). Furthermore, we also observed BmNPV-resistant strain had a much higher expression level of GDAP in midguts (Fig. 4H), and this might explain the higher resistance against BmNPV than susceptible strain. HSP60 is well known as a chaperon in that facilitates protein folding directly [37]. Vabulas et al. reported that endocytosed HSP60 could use Toll-like receptor 2 (TLR2) and TLR4 to activate the Toll/interleukin-1 receptor signaling pathway in innate immune cells [38]. Furthermore, HSP60 was determined to have the ability of binding to the 3′-UTR of the *Murine hepatitis virus* genome [39], and the contribution of HSP60 in anti-apoptotic program was confirmed in tumors *in vivo* [40]. The notable higher expression level (approximate 300 fold) of HSP60 in midguts of BmNPV-susceptible strain than in resistant strain (Fig. 4J) indicates its important role in BmNPV infection. The regulation of the five apoptosis-related genes (GDAP, PHB2, HSP60, CRT and RCX1) observed in this study is in congruent with their potential roles in response to virus infection, for example, up-regulation of the pro-apoptosis GDAP (Fig. 4H), while down-regulation of the other three anti-apoptosis genes (Fig. 4I, J and K), which in turn supported our hypothesized model below.

Ribosomes are important components of protein translation, and they contain a structure called ribosomal stalk [41], which forms a lateral protrusion from the ribosome and is composed of acidic proteins P0, P1, and P2 in eukaryotic cells [42]. Ribosomal P0 protein (RP0) functions as a scaffold for the stalk structure by interacting with 28S rRNA, and promotes Potato Virus A (PVA) infection of *Nicotiana benthamiana* by regulating PVA RNA expression [43]. We demonstrated the binding of BmNPV to RP0 of *B. mori in vitro* and determined its relative expression level in BmNPV susceptible and resistant strains of *B. mori* here, which (Fig. 4D) indicated potential role of RP0 in BmNPV infection process.

In recent years, arginine kinase (AK) has been studied not only in insects [44] but also in shrimps [45]. AK is a phosphagen kinase catalyzing the reversible transfer of the phosphoryl group of ATP to arginine yielding ADP and phosphoarginine, and has allergenic potential contributing to allergies against silkworm [46]. Injection of AK into *Litopenaeusvannamei* increased the mortality of shrimp infected with white spot syndrome virus (WSSV) indicating the involvement of AK in WSSV infection [47]. Kang et al. made a conclusion that AK was involved in the antiviral process of *B. mori* larvae against BmNPV infection by determining the relative expression level in BmNPV susceptible and resistant strains of *B. mori* [48]. Based on our results, the interaction between BmAK and BmNPV *in vitro* was confirmed by virus overlay assay, and the significantly higher expression of BmAK in susceptible strain (Fig. 4C) suggested its role in the infection process of BmNPV.

**Figure 5:**
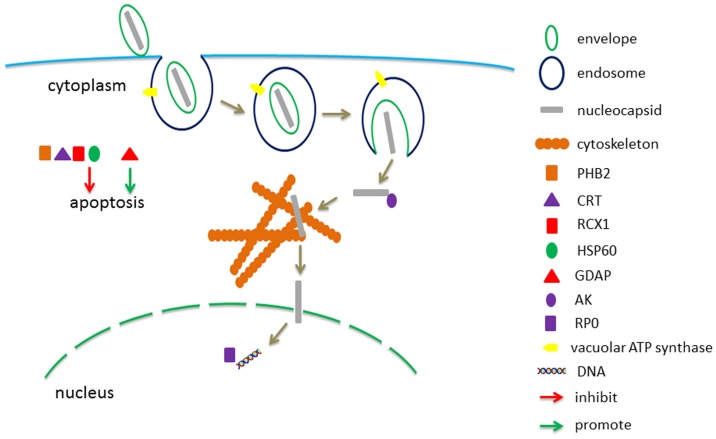
Hypothesized roles of the identified BmNPV-binding proteins of *B. mori* in the virus infection process. The envelope contained BmNPV nucleocapsid binded to the cytomembrane, then endocytosis was triggered, and vacuolar ATP synthase catalytic subunit A and subunit B on the endosome membrane could promote the fusion of the envelope and endosome to release the nucleocapsid into the cytoplasm. The released nucleocapsid was transported into the nucleus with the assistance of cytoskeleton (Actin, PGK and En). The virus DNA was released in the nucleus, and RP0 could regulate RNA expression of BmNPV. In the infection process, GDAP would trigger apotosis to inhibit BmNPV infection, while PHB2, CRT, RCX1 and HSP60 had the opposite effects.

Based on the results and analysis above, we hypothesize the roles of these binding proteins in the process of crossing BmNPV through silkworm midgut cells. The BmNPV nucleocapsid contained envelope binds to the cytomembrane, and endocytosis is triggered. Then ATP-A and ATP-B on the endosome membrane promote the fusion of the envelope and endosome to release the nucleocapsid into cytoplasm. The released nucleocapsid is transported into the nucleus by the assistance of cytoskeleton (Actin, PGK and En). The virus DNA is released in the nucleus, and RP0 could regulate RNA expression of BmNPV. In the infection process, GDAP would trigger apoptosis to inhibit BmNPV infection, while PHB2, CRT, RCX1 and HSP60 had the opposite effects (Fig. 5).

As far as we know, this is the first report that identifies BmNPV binding proteins on proteomic and transcriptional level. Although the functions of the proteins are based on literature illustrations, we believe that these deductions are reasonable. Taken together, our work provides a novel direction to research the molecular mechanisms of the interactions between silkworms and BmNPV.

## Materials and Methods

### Insects and virus

P50, a standard reference silkworm strain, was maintained in the Key Laboratory of Sericulture, Anhui Agricultural University. A35, a BmNPV resistant strain, was also maintained in our laboratory. The resistant levels of these two strains against BmNPV were determined before [49]. The first three instars larvae were reared on fresh mulberry leaves or artificial diet at 27±1°C, 75±5% of relative humidity with 12 hours day/night cycles and the last two instars larvae were reared at 24±1°C, and the same relative humidity and photoperiod as above.

BmNPV T3 strain was maintained in our laboratory. The occlusion body (OB) of T3 strain was obtained from the haemolymph of infected larvae and was purified by repeated and differential centrifugation following the previously published protocol [50], and the concentration of the virus (OB/ml) was determined using haemocytometer. The budded virus (BV) was supplied by Jiangsu University.

### Extraction of total proteins

Fifth instar larvae were dissected at the third day after molting, and the midguts were frozen with liquid nitrogen and then pulverized. Total protein extraction was performed as described by the Instruction Manual of ReadyPrep Protein Extraction Kit (Bio-Rad). Briefly, 100 mg pulverized midgut was added into a 2 mL microcentrifuge tube containing 1 mL of 2-D Rehydration/Sample Buffer 1 with 10 µL of 200 mM ReadyPrep tributylphosphine reducing agent, 10 µL of 40% (w/v) ampholyte (Bio-Rad, Bio-Lyte 3/10) and 10 µL of 100 mM phenylmethanesulfonyl fluoride (PMSF), and the sample was sonicated until lysis was complete on ice. After a centrifugation(Hettichi MIKRO 220R, 1195A) at 16,000×*g* for 30 min at 4°C the supernatant was transferred to a clean tube. The protein sample was characterized by 12% SDS-PAGE, and the concentration was determined with Bradford using BSA as standard.

### Extraction of lipid-associated membrane proteins

Lipid-associated membrane proteins from larvae midgut were isolated as described by Dickerson et al. [51] with some modifications. Briefly, 100 mg of pulverized midgut was added into a 2 mL microcentrifuge tube containing 1 mL of ice-cold Triton X-114 extraction buffer[2.0%(v/v) triton X-114, 300 mM NaCl, 20 mM Tris-HCl (pH7.5), 1 mM PMSF], and the sample was sonicated until lysis was complete on ice. After a centrifugation at 16,000×*g* for 10 min at 4°C, the supernatant was transferred into a clean tube on ice. The supernatant was incubated in a 30°C water-bath for 5 min and was layered onto a sucrose cushion of equal volume[6.0% (w/v) sucrose, 150 mM NaCl, 0.06%(v/v) Triton X-114, 10 mM Tris-HCl (pH 7.5)] in a clean tube, and warmed in 30°C water-bath for 5 min followed by a centrifugation at 300×*g* for 3 min at room temperature (RT). The aqueous phase was transferred to another tube and treated again as described above, and the remaining detergent phase was saved for later use. Both the lipoproteins and hydrophilic proteins were precipitated by adding nine times volume of ice-cold acetone. The precipitated proteins were suspended in 10 mM Tris-HCl (pH7.5) for concentration determinations with Bradford.

### Sodium dodecyl sulfatepolyacrylamide gel electrophoresis(SDS-PAGE) and two-dimensional electrophoresis (2-DE)

For SDS-PAGE, 5×protein loading buffer (50 mM Tris-HCl pH 8.0, 250 mMDTT, 5% SDS, 50% Glycerol, 0.04% Bromophenol Blue) was added to the total protein sample and the purified BmNPV particles. Samples were boiled for 10 min and loaded onto a sodium dodecyl sulfate (SDS) polyacrylamide gel. Electrophoresis was performed in the Bio-Rad Mini-protean Tetra system. In the procedure of 2-DE, the first step, isoelectric focusing (IEF), was performed in Bio-Rad protean IEF cell. Briefly, samples (125 µL/strip) were loaded on ReadyStrip IPG strips (7 cm) with linear pH 3–10 gradients. After active rehydration (50V) for 14 h, IEF was performed following a voltage step-gradient (250V linear for 1 h, 500V rapid for 1 h, 4000V linear for 3 h, and 4000V rapid for 20,000 V·h) at 20°C, with a maximum current of 50 mA/strip. Before the second step (SDS-PAGE), the IPG strips were first equilibrated for 15 min in a solution containing 6 M urea, 2% (w/v) SDS, 0.375 M Tris-HCl pH 8.8, 20% (v/v) glycerol, and 2% (w/v) DTT, and then for 15 min in the same solution, substituting DTT with 2.5% (w/v) iodoacetamide. SDS-PAGE was carried out in 1 mm-thick 12% SDS polyacrylamide gels. Gels were run under a constant current of 20 mA until the bromphenol blue dye front migrated to the bottom.

### Virus overlay assay (far-western blot)

After electrophoresis, proteins in a gel were transferred onto a polyvinylidenedifluoride (PVDF) membrane (Millipore) following a Biometre wet blotting procedure (150 mA for 120 min). To locate potential virus-binding proteins from SDS polyacrylamide gels or 2-DE gels for identification, the parallel gel was stained with Coomassie brilliant blue G-250.

The far-Western blot was performed as described by Wu et al. [52], with some modifications. Proteins on the membrane were denatured and renatured in AC buffer (100 mM NaCl, 20 mMTris-HCl pH 7.6, 0.5 mM EDTA, 10% glycerol, 0.1% Tween-20, 2% no-fat milk, 1 mM DTT) by gradually reducing the guanidine-HCl concentration. Briefly, the membrane was incubated in the AC buffer containing 6 M guanidine-HCl for 30 min at RT, and then washed with the AC buffer containing 3 M guanidine-HCl for 30 min at RT. This is followed by washing with the AC buffer containing 0.1 M and no guanidine-HCl AC buffer at 4°C, for 30 min and 1 h, respectively, and then blocked for 30 min at RT in blocking buffer [PBST (137 mM NaCl, 2.7 mM KCl, 10 mM Na_2_HPO_4_, 2 mM KH_2_PO_4_, pH7.5, 0.05% (v/v) Tween-20), 5% (w/v) no-fat milk]. The membrane was subsequently incubated overnight in binding buffer (100 mM NaCl, 20 mM Tris-HCl pH 7.5, 0.5 mM EDTA, 10% (v/v) glycerol, 2% (w/v) no-fat milk, and 1 mM DTT) containing purified BmNPV particles (5 mg/mL). After washed three times for 10 min each in PBST, the membrane was incubated with monoclonal antibodies (MAbs) against baculovirus gp64 (Santa Cruz) with a dilution of 1∶500 in blocking buffer for 3 hours at RT. After washing as above, antigen-antibody complexes were detected with a horseradish peroxidase (HRP)-conjugated goat anti-mouse secondary antibody (1∶5000 dilution) in blocking buffer for 1.5 hours at RT. After another series of washes, immobilized conjugates on the membrane were visualized in HRP substrate solution (Tiangen). For the negative control, the membrane was incubated overnight in binding buffer without BmNPV particles, followed by antibody incubation.

### Protein identification

Comparisons between the stained gels and the results of far-western blot experiments on the membrane allowed the unambiguous selection of protein bands from SDS polyacrylamide gels or spots from 2-DE gels for MALDI-TOF/TOF MS analysis on ABI 4800 Plus MALDI TOF/TOF Analyzer. Selected protein bands or spots were cut out from gels, and the pieces were destained with 100 mM NH_4_HCO_3_ in 30% (v/v) acetonitrile for 15 min. The liquid phase was removed, and the gel pieces were completely lyophilized, followed by swelling in a digestion buffer containing 10ng/ml trypsin (Promega) overnight at 37°C. The digested peptides were lyophilized and dissolved in 20% (v/v) acetonitrile. One microliter of the sample was added to the target site and taken into the analyzer after dried naturally. The peptide sequences obtained from MALDI-TOF/TOF MS were searched against the protein sequences from NCBInrmetazoa using the Mascot algorithm (http://www.matrixscience.com). The search parameters were set as follows: Type of search, MS/MS Ion Search; enzyme, trypsin; fixed modifications, carbamidomethyl (C); variable modifications, Acetyl (Protein N-term), Deamidated (NQ), Dioxidation (W), Oxidation (M); Mass values, Monoisotopic; Protein Mass, Unrestricted; mass tolerances for MS/MS were 100 ppm and 0.5 Da; max missed cleavages, one. Protein identification was accepted when the matching scores were significant at P<0.05, as based on the Mowse score (Matrix Science, London, UK).

### RNA isolation and cDNA synthesis

Based on the reported time course of viral proliferation and expression of NPV responsive genes in fat body and haemocytes of susceptible and resistant strains [53], silkworm from the infected and control groups were dissected at 48 hour post inoculation (h.p.i), and the midguts were frozen with liquid nitrogen and then pulverized. One hundred milligram of midgut was added into a RNAase free microcentrifuge tube containing 1000 µL TRIZOL Reagent (Life Technologies), then homogenized with a pellet pestle motor. Total RNA was extracted from the midguts using TRIZOL Reagent according to the manufacturer's instructions of Invitrogen. The ratios of A260/280 and the concentrations for the RNA samples were determined by NanoDrop 2000 spectrophotometer (Thermo Scientific).

Total RNA samples were treated with RT reagent kit with gDNA Eraser (TaKaRa) to remove genomic DNA and the first strand cDNA was synthesized according to the manufacturer's instructions. Briefly, 2.0 µL of 5×gDNA Eraser buffer, 1.0 µL of gDNA Eraser, and 1.0 µg of total RNA were mixed in a 200 µL PCR tube and added up RNase Free dH_2_O to 10 µL, and then incubated at room temperature for 5 minutes. Four micro liter of 5×PrimeScript buffer, 1.0 µL of PrimeScript RT Enzyme Mix I, and 1.0 µL of RT Primer Mix were added to the previous tube, then added up to 20 µL with RNase Free dH_2_O. The mix was incubated at 37°C for 15 minutes followed by 85°C for 5 seconds and stored at −20°C for later use.

### qPCR analysis

qPCR was carried out in a 25 µL reaction mix containing 12.5 µL of SYBR Premix Ex Taq (TaKaRa), 1 µL of 1∶5 diluted cDNA template, 1 µL of each of the primers (10 µM) and 9.5 µL ddH2O. The thermal cycling profile consisted of initial denaturation at 95°C for 30 s and 40 cycles at 95°C for 5 s, 60°C for 30 s, and 72°C for 20 s. Relative expression levels were calculated using the 2−ΔΔCt method where ΔΔCt = ΔCt sample−ΔCt reference following the previously published protocol [54]. PCR reactions were performed on Bio-Rad CFX96TM Real-Time System using SYBR Green to detect dsDNA synthesis. PCR amplification was performed in triplicate wells. The *B. mori* ribosomal protein s3 gene (*Bmrps3*) was set as an internal control. Data were normalized using *Bmrps3*, and the statistical analysis was conducted using ANOVA and LSD aposteriori test (*P*<0.01).

## Supporting Information

S1 Figure **Details of ATP-A (band) identified by MALDI-TOF/TOF MS.**
(TIF)

S2 Figure **Details of Actin identified by MALDI-TOF/TOF MS.**
(TIF)

S3 Figure **Details of RP0 identified by MALDI-TOF/TOF MS.**
(TIF)

S4 Figure **Details of PHB2 identified by MALDI-TOF/TOF MS.**
(TIF)

S5 Figure **Details of GDAP identified by MALDI-TOF/TOF MS.**
(TIF)

S6 Figure **Details of ATP-A (spot) identified by MALDI-TOF/TOF MS.**
(TIF)

S7 Figure **Details of HSP60 identified by MALDI-TOF/TOF MS.**
(TIF)

S8 Figure **Details of ATP-B identified by MALDI-TOF/TOF MS.**
(TIF)

S9 Figure **Details of Crt identified by MALDI-TOF/TOF MS.**
(TIF)

S10 Figure **Details of En identified by MALDI-TOF/TOF MS.**
(TIF)

S11 Figure **Details of PGK identified by MALDI-TOF/TOF MS.**
(TIF)

S12 Figure **Details of AK identified by MALDI-TOF/TOF MS.**
(TIF)

S13 Figure **Details of RCX1 identified by MALDI-TOF/TOF MS.**
(TIF)
