# Supplementary figures and images for: A Hypothetical Model of Crossing Bombyx mori Nucleopolyhedrovirus through Its Host Midgut Physical Barrier
Source: PLoS One. 2014 Dec 12;9(12):e115032. doi: 10.1371/journal.pone.0115032 (PMC4264868; doi:10.1371/journal.pone.0115032)

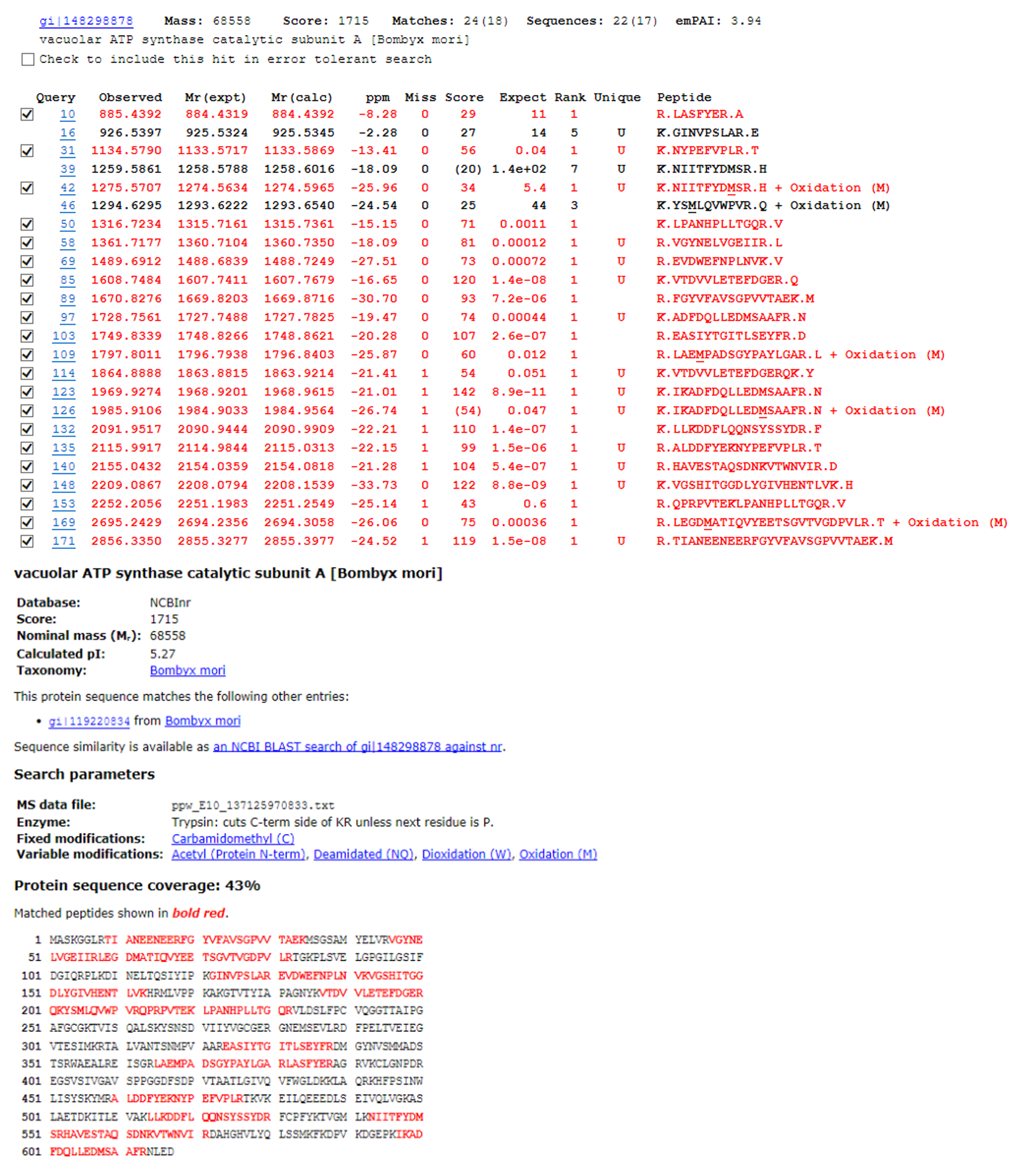

Supplement: S1 Figure — Details of ATP-A (band) identified by MALDI-TOF/TOF MS. (TIF) [file pone.0115032.s001.tif]

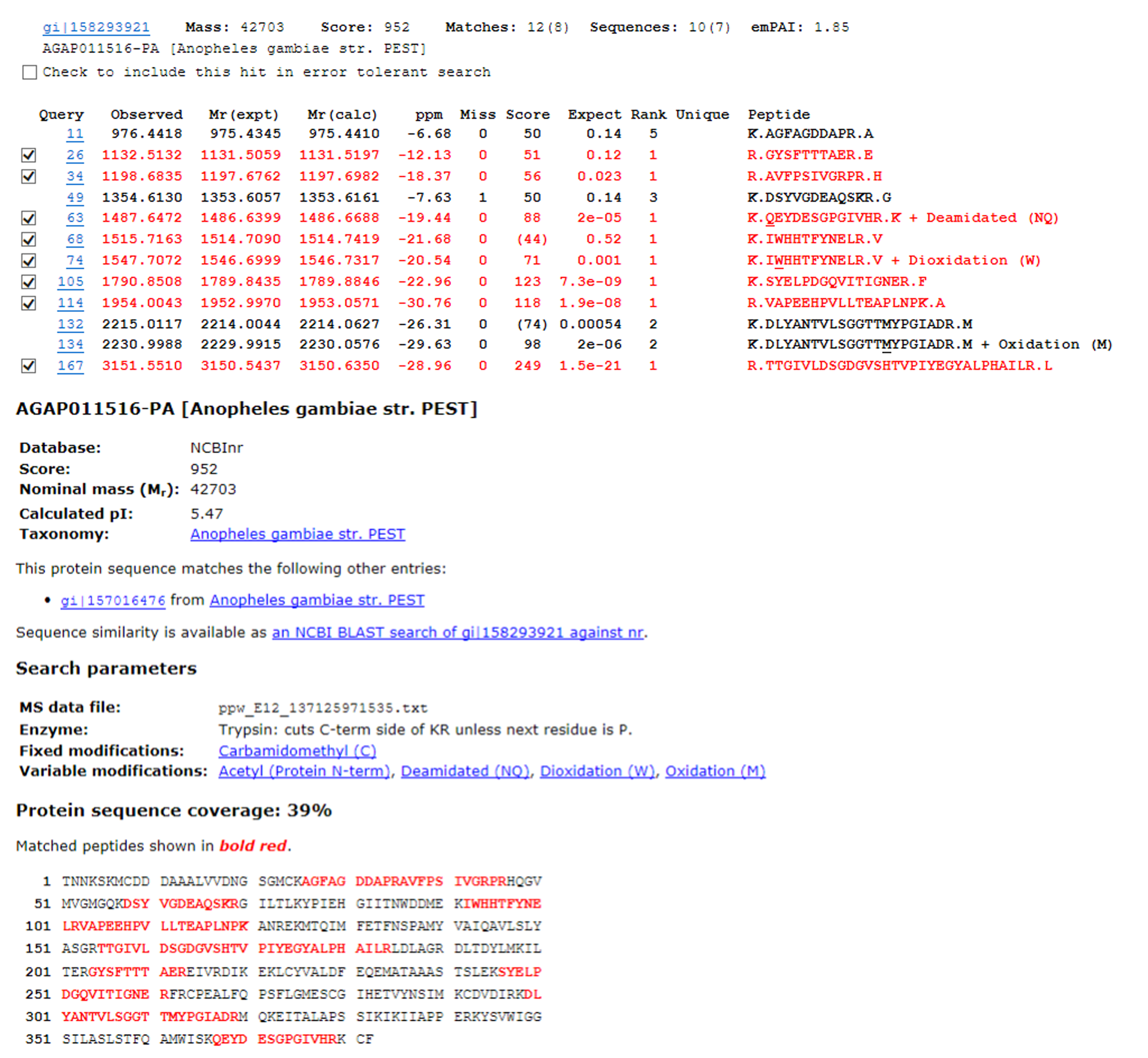

Supplement: S2 Figure — Details of Actin identified by MALDI-TOF/TOF MS. (TIF) [file pone.0115032.s002.tif]

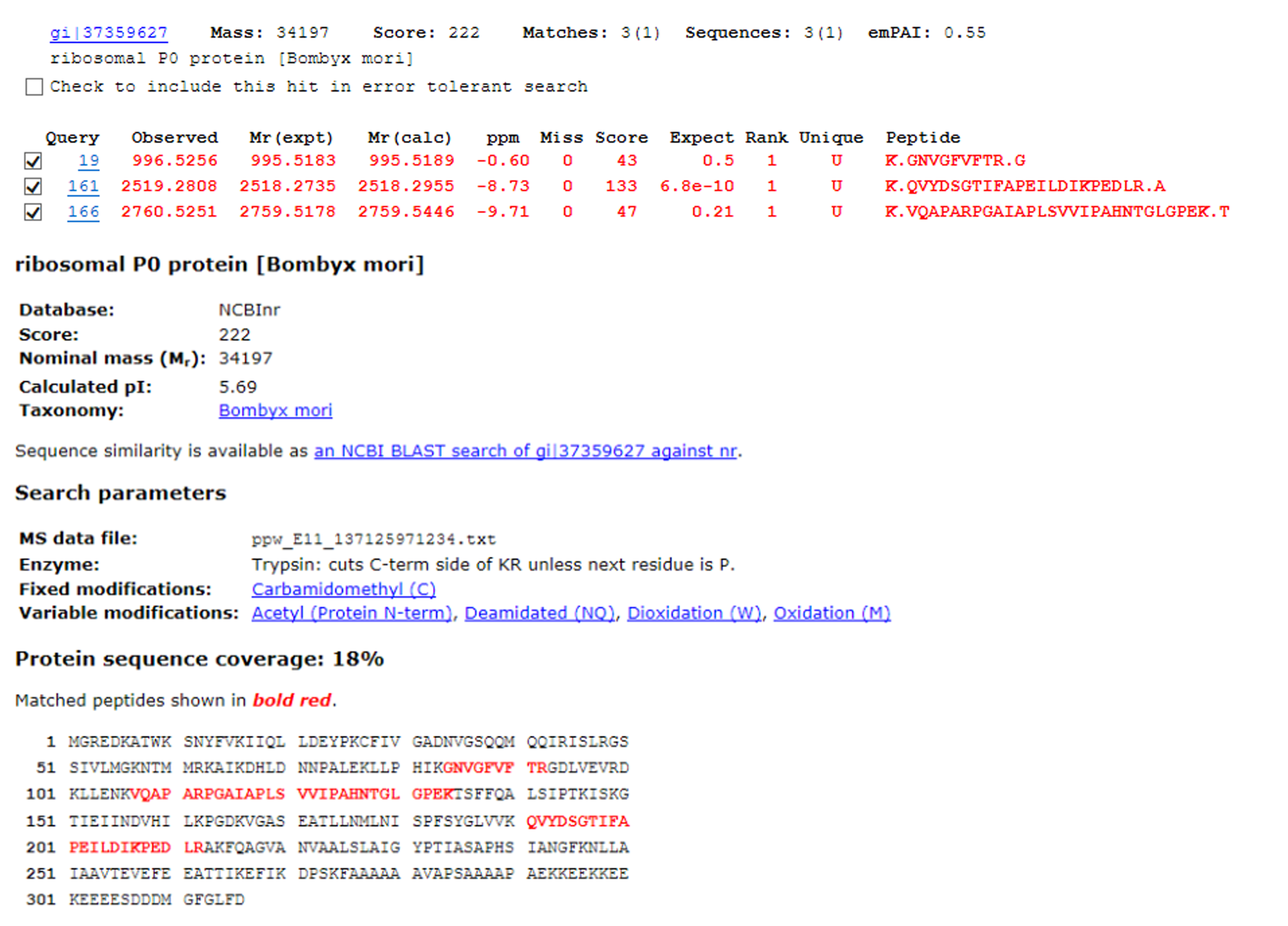

Supplement: S3 Figure — Details of RP0 identified by MALDI-TOF/TOF MS. (TIF) [file pone.0115032.s003.tif]

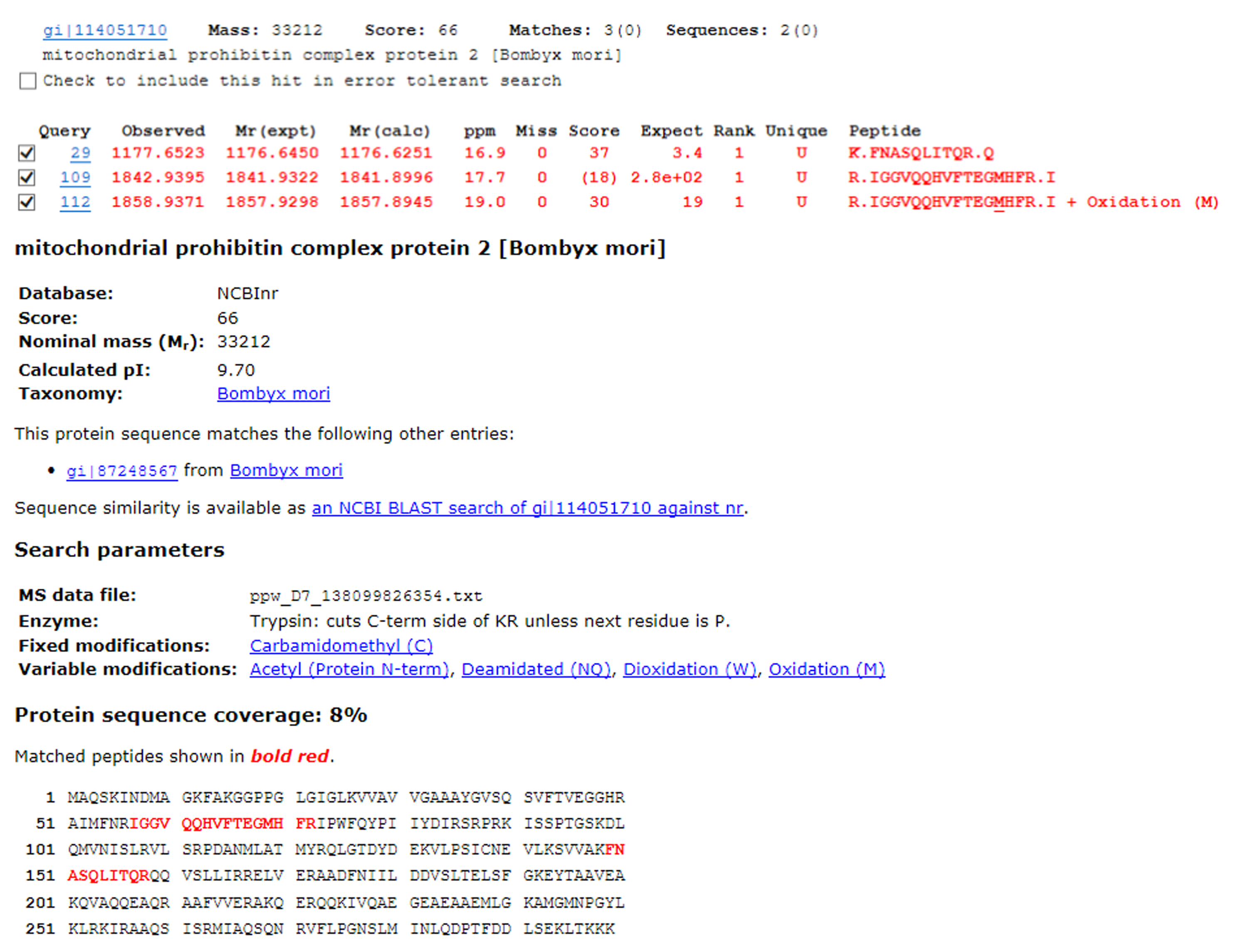

Supplement: S4 Figure — Details of PHB2 identified by MALDI-TOF/TOF MS. (TIF) [file pone.0115032.s004.tif]

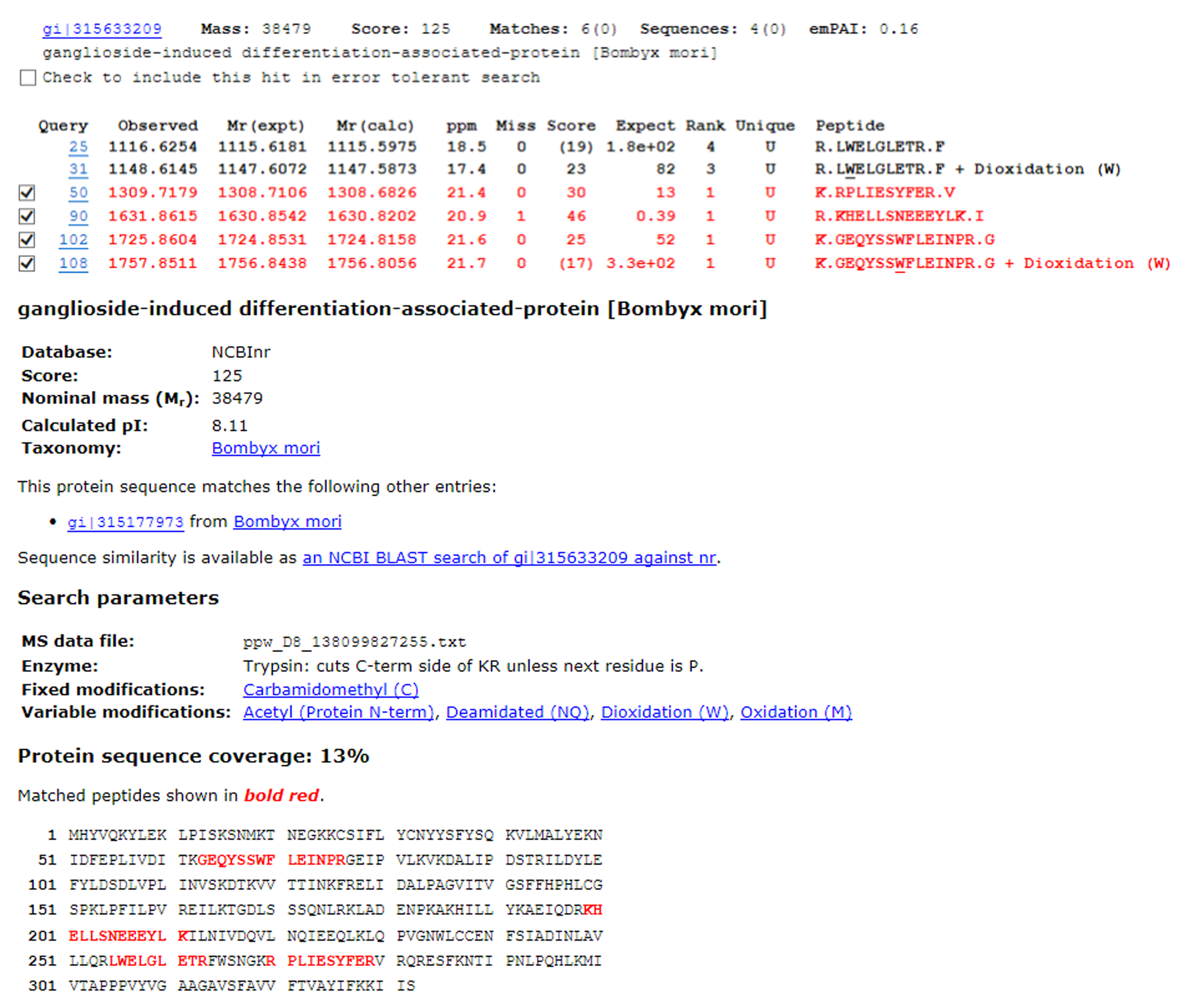

Supplement: S5 Figure — Details of GDAP identified by MALDI-TOF/TOF MS. (TIF) [file pone.0115032.s005.tif]

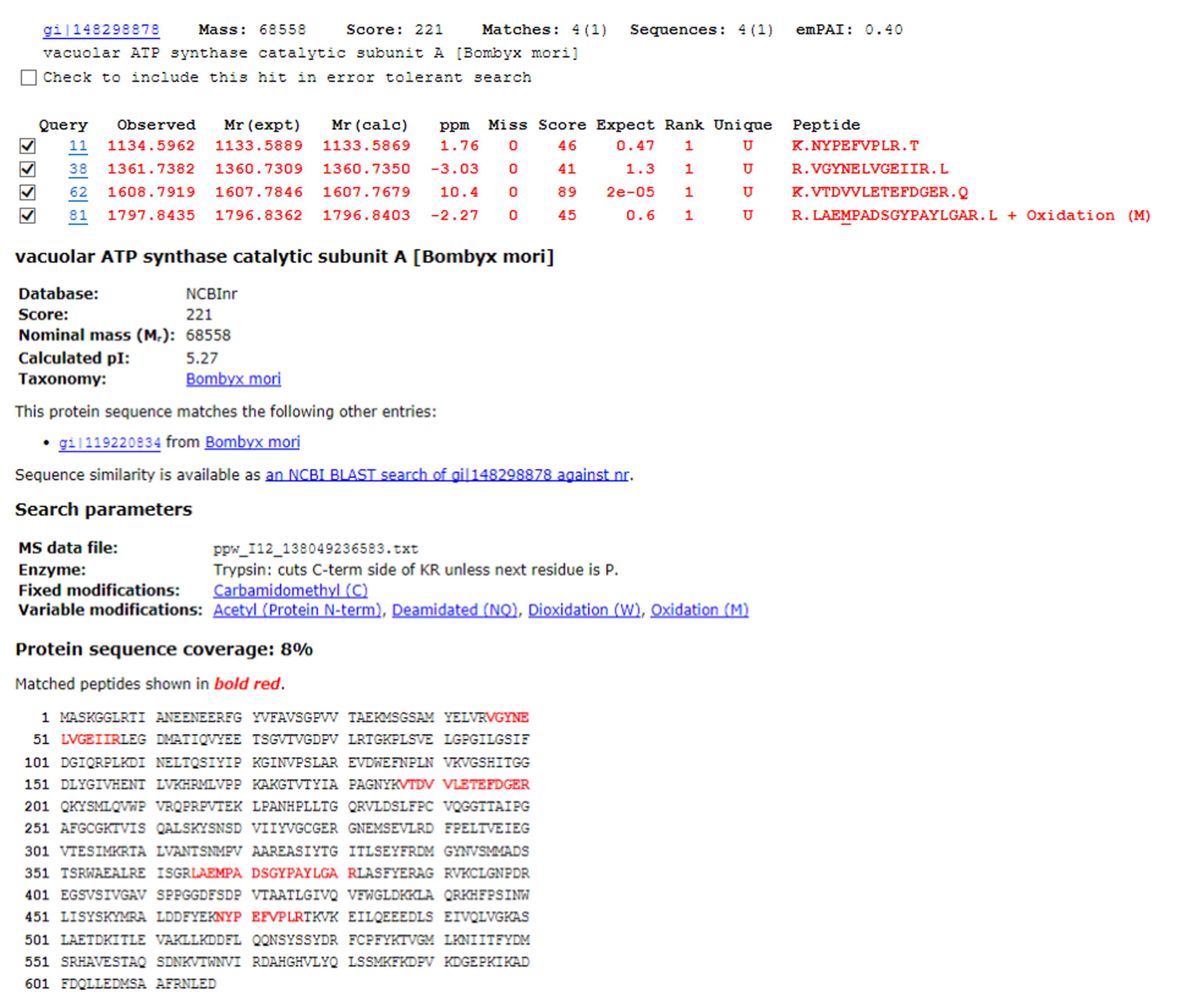

Supplement: S6 Figure — Details of ATP-A (spot) identified by MALDI-TOF/TOF MS. (TIF) [file pone.0115032.s006.tif]

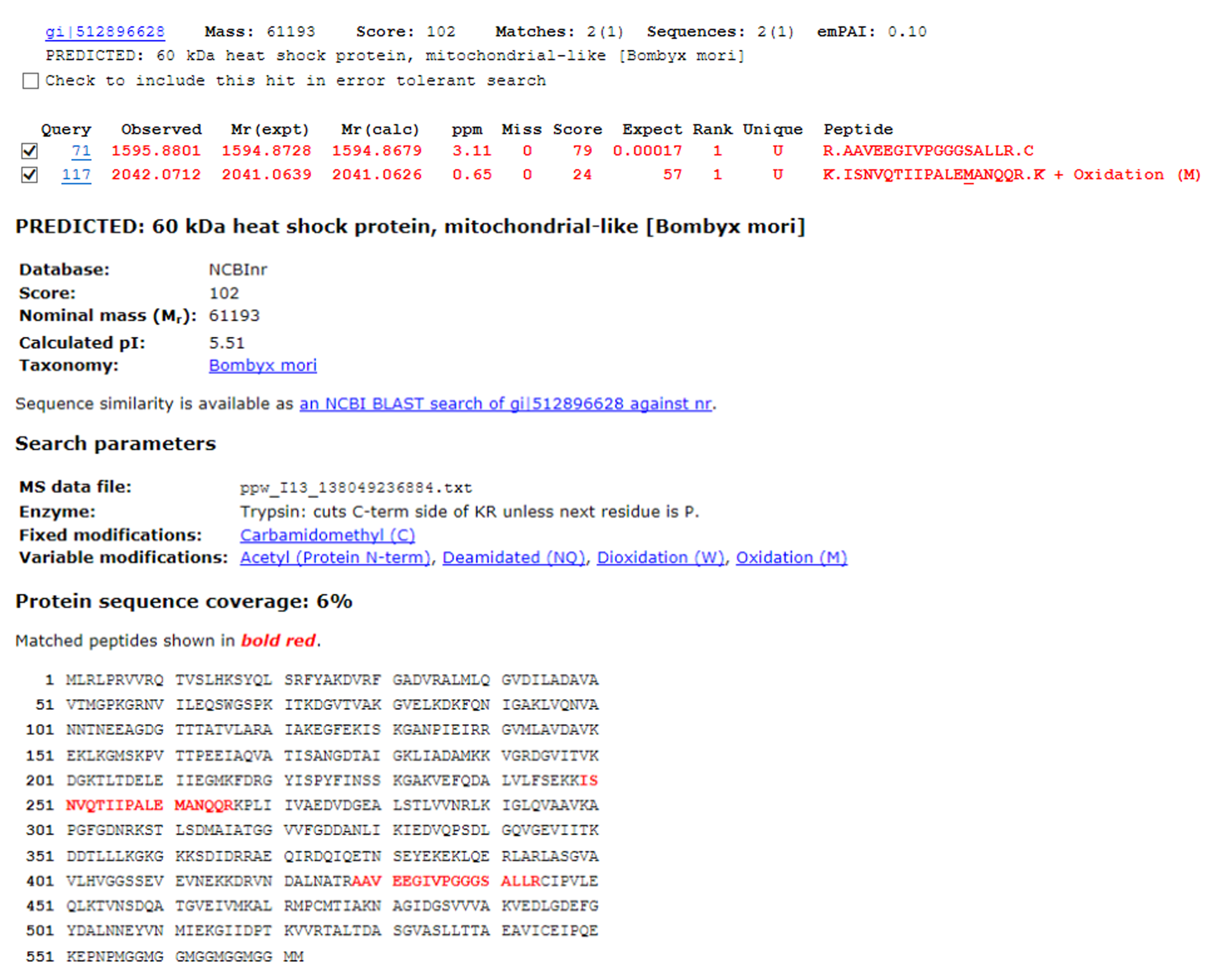

Supplement: S7 Figure — Details of HSP60 identified by MALDI-TOF/TOF MS. (TIF) [file pone.0115032.s007.tif]

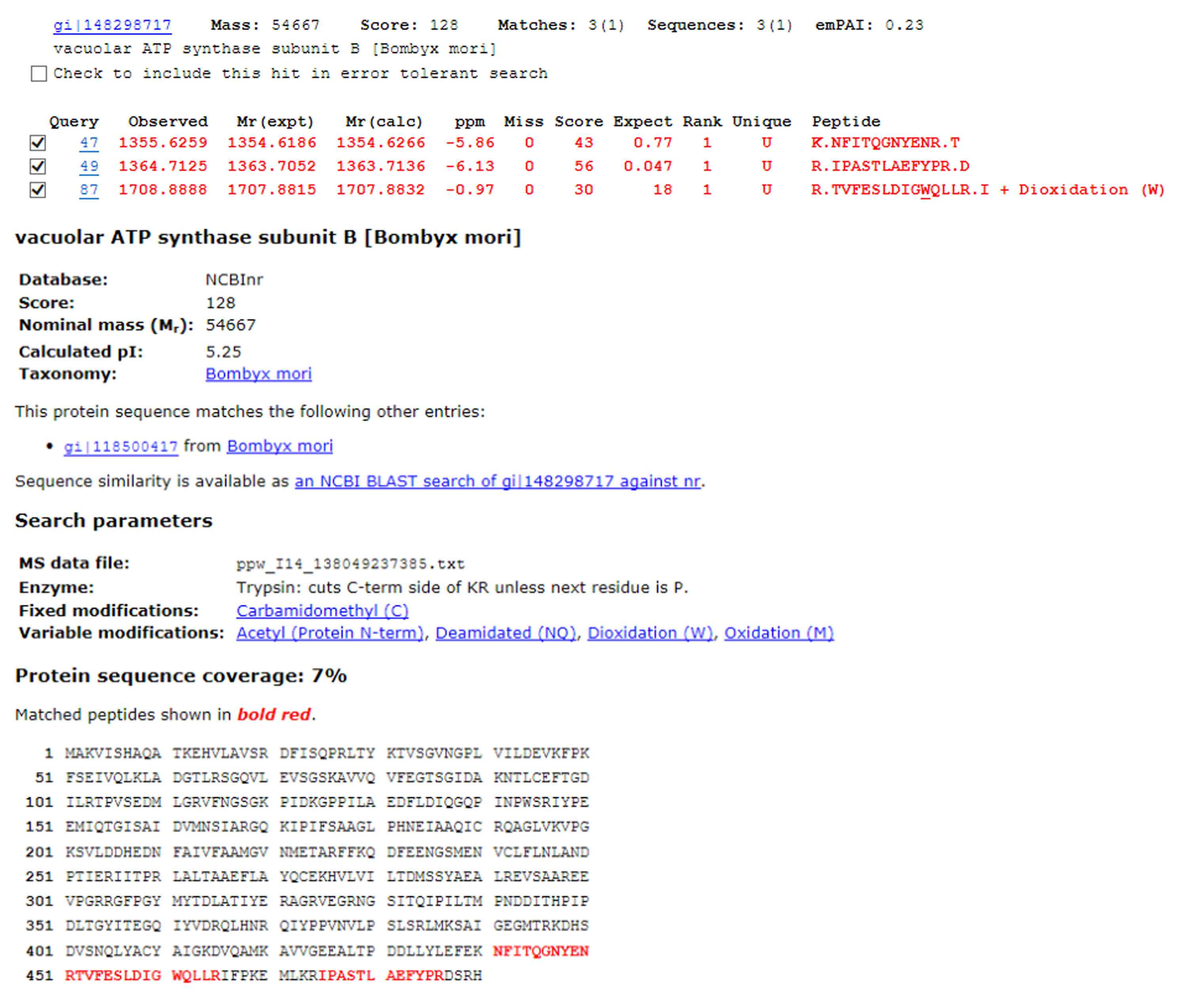

Supplement: S8 Figure — Details of ATP-B identified by MALDI-TOF/TOF MS. (TIF) [file pone.0115032.s008.tif]

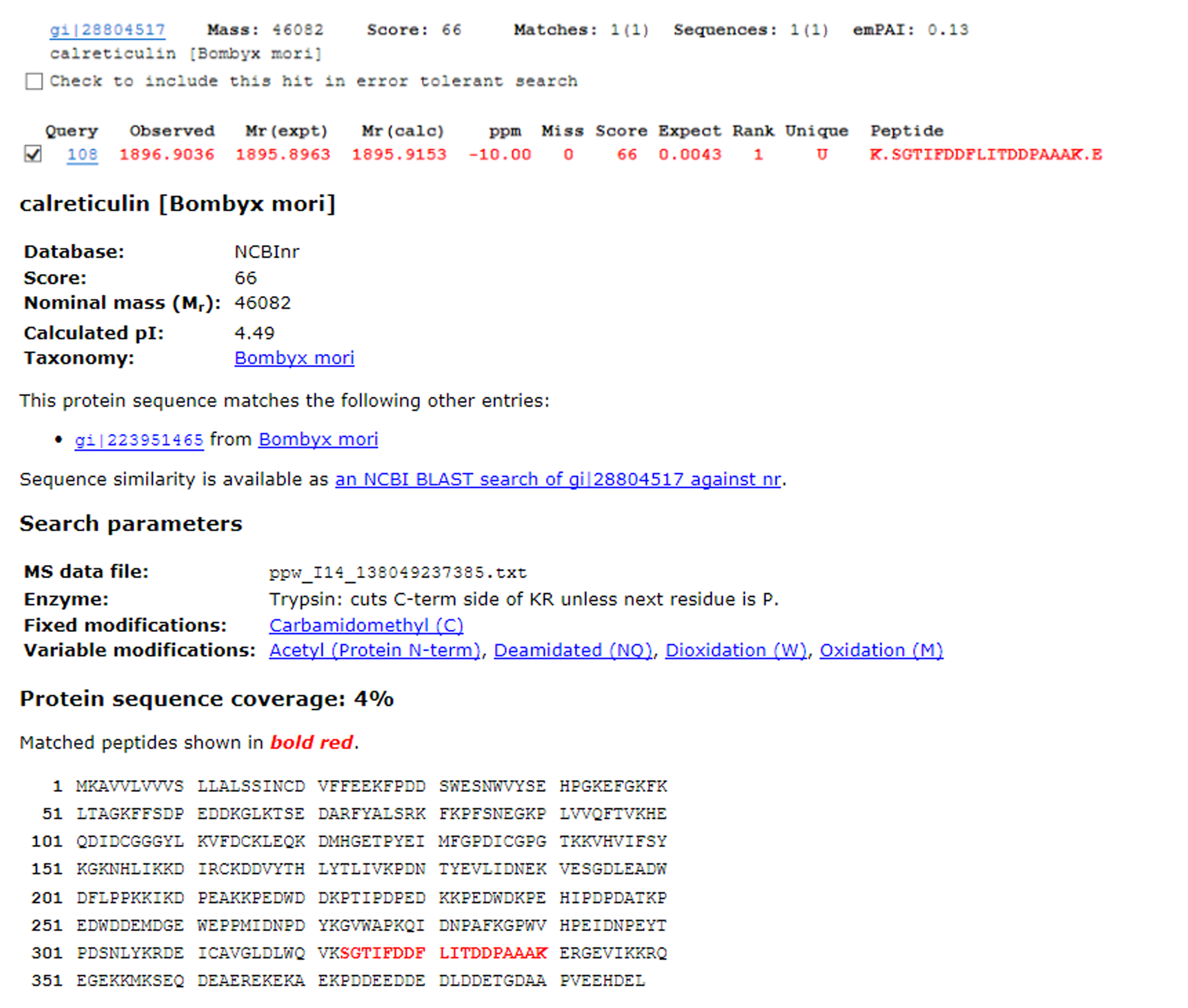

Supplement: S9 Figure — Details of Crt identified by MALDI-TOF/TOF MS. (TIF) [file pone.0115032.s009.tif]

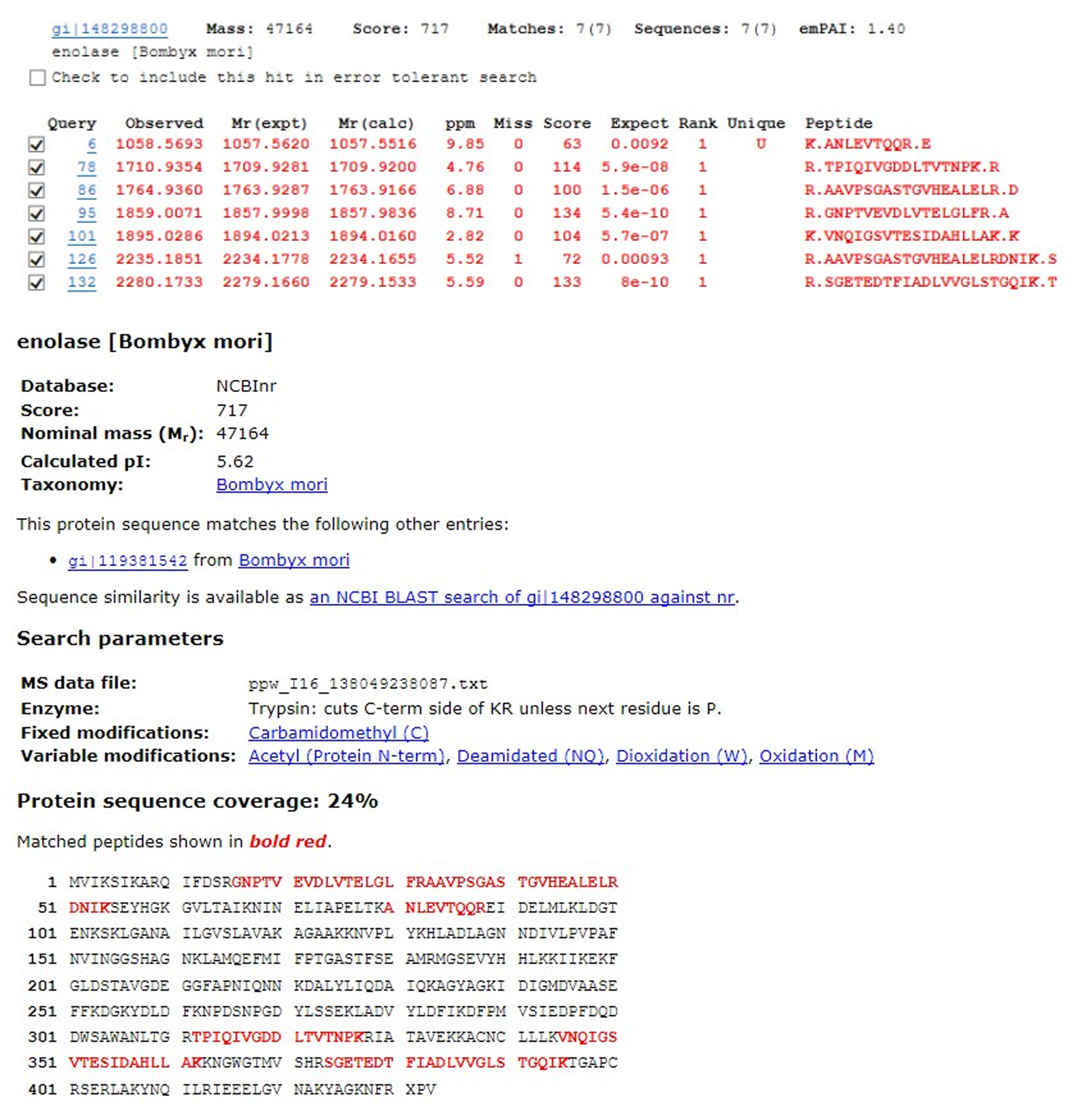

Supplement: S10 Figure — Details of En identified by MALDI-TOF/TOF MS. (TIF) [file pone.0115032.s010.tif]

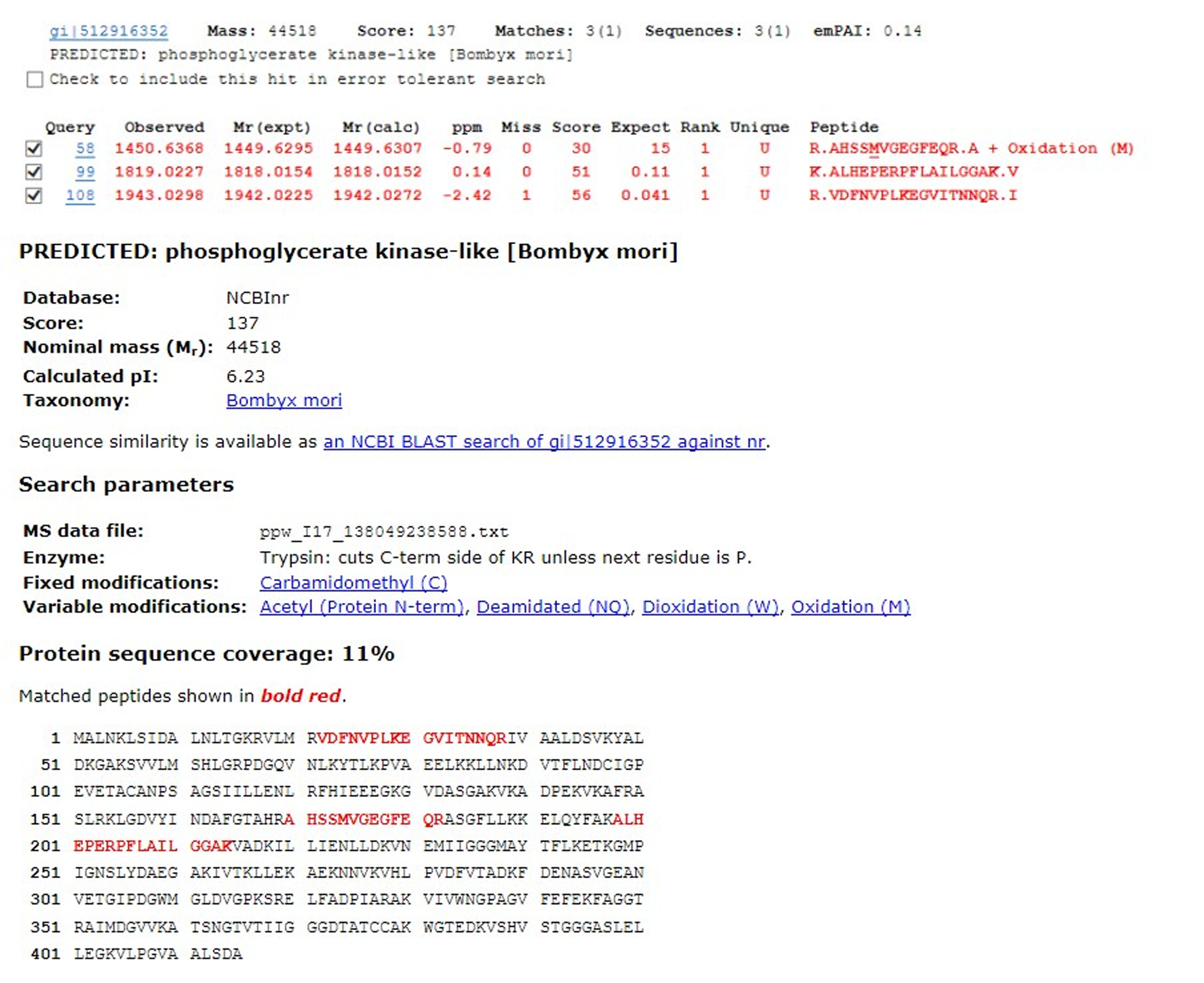

Supplement: S11 Figure — Details of PGK identified by MALDI-TOF/TOF MS. (TIF) [file pone.0115032.s011.tif]

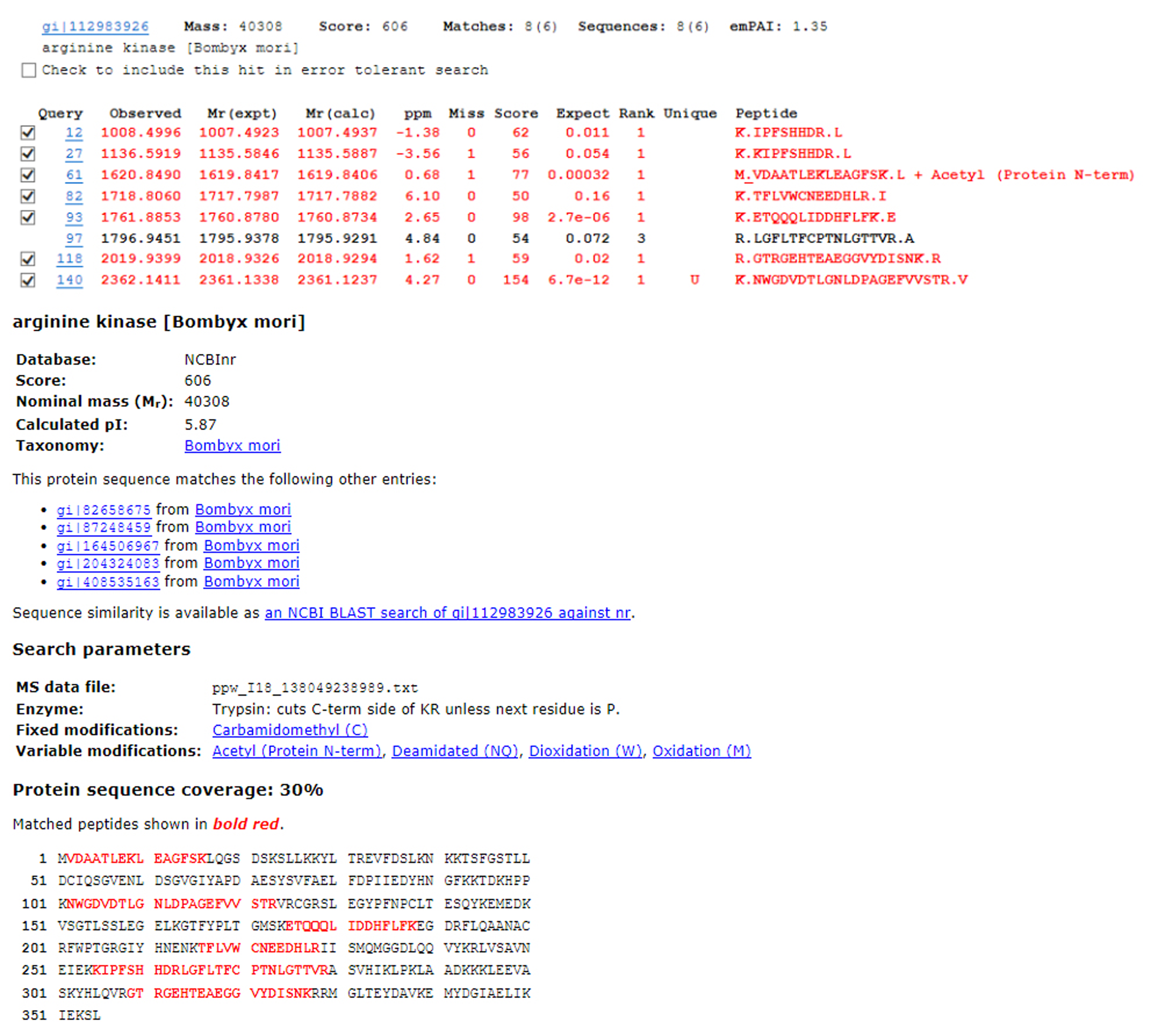

Supplement: S12 Figure — Details of AK identified by MALDI-TOF/TOF MS. (TIF) [file pone.0115032.s012.tif]

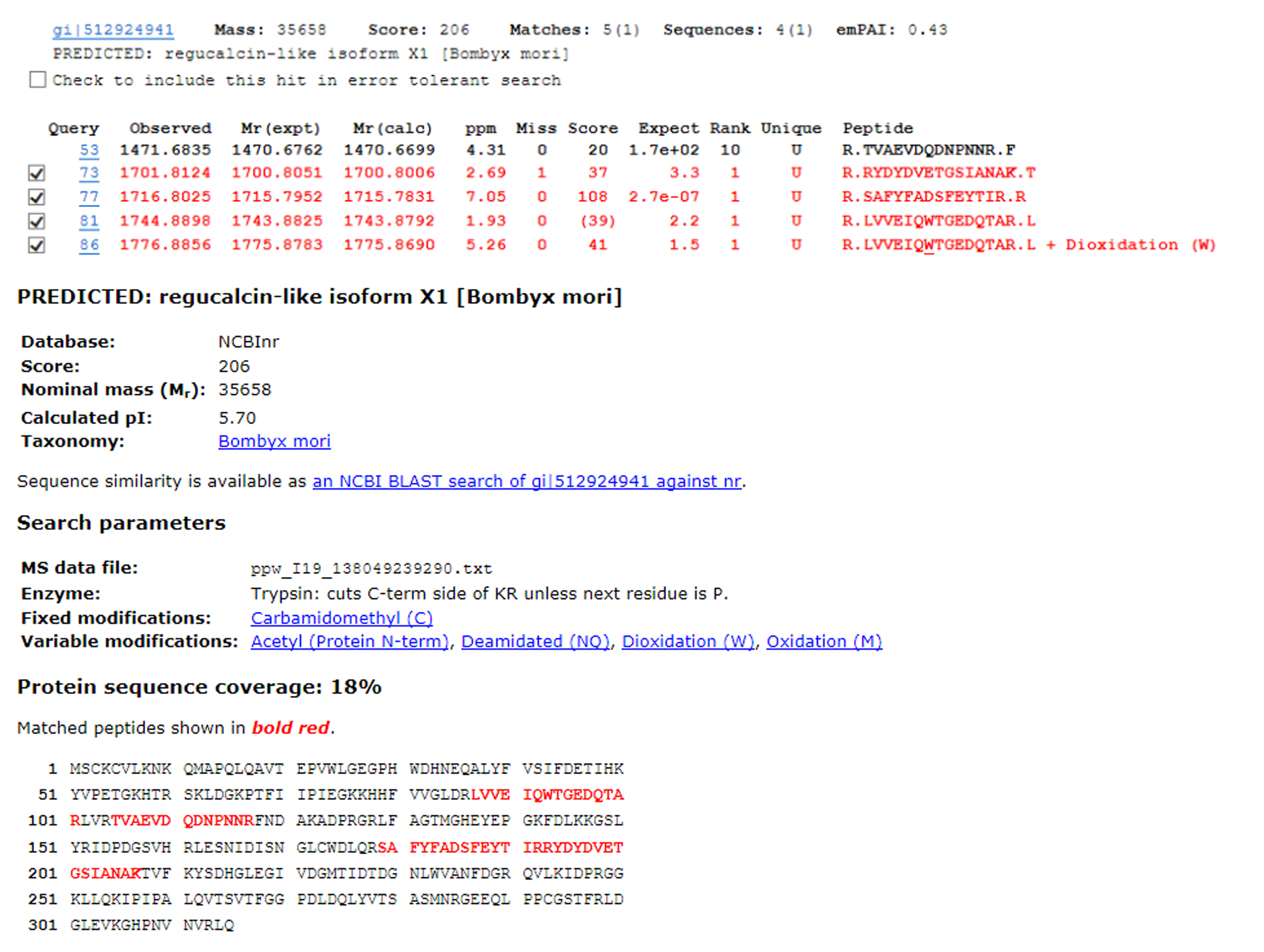

Supplement: S13 Figure — Details of RCX1 identified by MALDI-TOF/TOF MS. (TIF) [file pone.0115032.s013.tif]
